# First evidence of “ancient deer” (cervid) in the late Miocene Bira Formation, Northern Israel

**DOI:** 10.1371/journal.pone.0185268

**Published:** 2017-11-01

**Authors:** Alexis Gabriel Rozenbaum, Dotan Shaked Gelband, Mordechai Stein, Henk K. Mienis, Rivka Rabinovich

**Affiliations:** 1 Geological Survey of Israel, 30 Malkhe Israel, Jerusalem, Israel; 2 Institute of Earth Sciences, The Hebrew University of Jerusalem, Jerusalem, Israel, Edmond J. Safra Campus, Givat Ram, Jerusalem, Israel; 3 Natural History Collections, The Hebrew University of Jerusalem, Edmond J. Safra Campus, Givat Ram, Jerusalem, Israel; Seconda Universita degli Studi di Napoli, ITALY

## Abstract

Despite the extensive geological and paleontological searches in the south Levant, no terrestrial fauna of late Neogene age was yet reported. Here, we report the first evidence of “ancient deer”–cervid in the late Miocene (Tortonian) lacustrine section of the Bira Formation at Hagal Stream, Jordan Valley, northern Israel. The section comprises rich assemblage of macrofauna fossils, mostly freshwater mollusks. The mammalian bone was discovered among the macrofauna fossils, and is described as an almost complete left humerus of an adult animal identified as an artiodactyls element probably of a cervid. This terrestrial mammal shares similar paleoenvironmental conditions with other contemporaneous localities, where cervids prevailed with bovids and other taxa. It appears that the freshwater lakes provided favorite habitat for the development of the cervids and possibly other mammals. The specific conditions of preservation of the cervid illuminate the role of post depositional processes (taphonomic constrains) in masking terrestrial fauna remains in the region. Accordingly, further efforts will be devoted to unveil the mute part of the southern Levant–east Mediterranean terrestrial faunistic realm at the end of the Miocene.

## 1. Introduction

**Fig 1:**
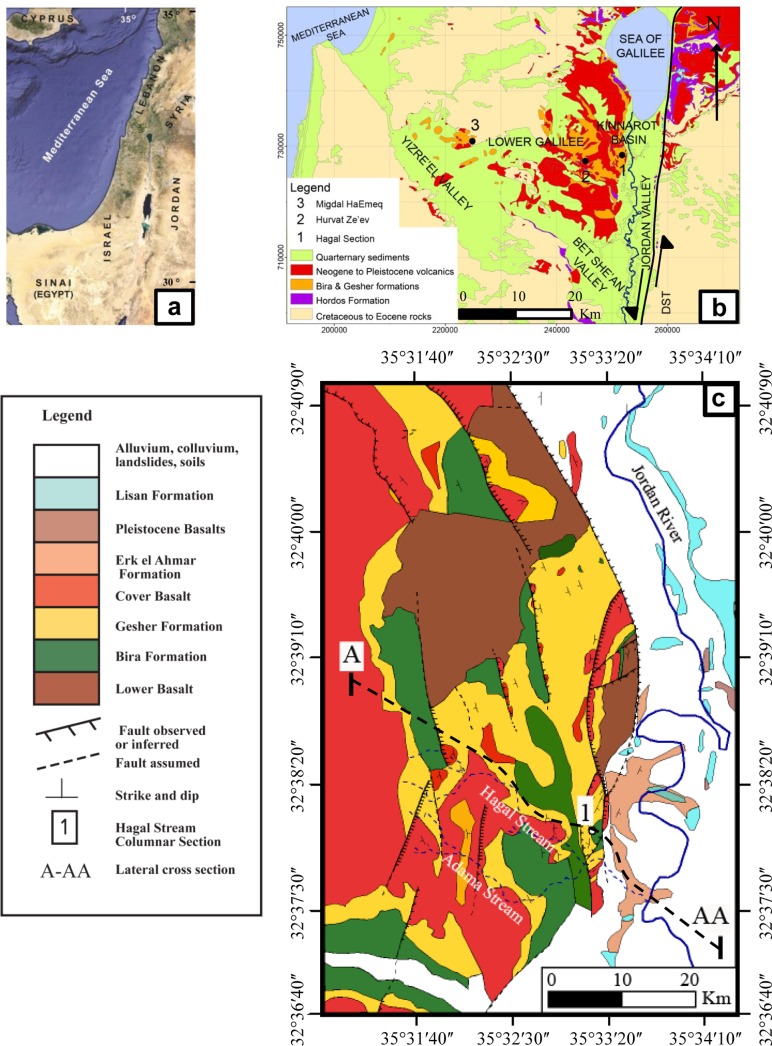
(a) Regional location map; (b) Simplified geological map of northern Israel (modified after [34]); (c) Geological map of the Hagal Stream on the Kinnarot Basin (modified after [17, 35]).

The Miocene-Pliocene periods were distinguished by significant tectonic deformation and magmatic activity throughout the Arabian and African plates [1–3]. The breakup of the Arabian-African continent was accompanied by the opening of the Red Sea, the formation of the Suez rift and the Dead Sea Transform (DST) [4, 5] (Fig 1A and 1B). The rifting process was accompanied by widespread magmatic activity, mainly eruption of alkali basalts [6 and references there]. At the early to middle Miocene (~ 20–10 Ma) the areas that extend now over the Lower Galilee- Yizre’el Valley and the Jordan Valley (Fig 1B) were characterized by a low relief with a large river systems draining from east to west and depositing sequences of fluvial/alluvial sediments (the Hordos Formation, e.g. [7–9]). During this time, extensive volcanic eruptions occurred over the Arabian plate producing in northern Israel and Golan Heights thick sequences of alkali basalts that comprise the Lower Basalt unit (spanning ~ 17.5–10 Ma -, see Fig 1B) (e.g. [7, 9–11]). At the same time the morphological basins and valleys of northern Israel deepened (e.g. [5, 7]). This was accompanied by a reversal in the direction of the drainage system towards the east where the Jordan rift valley, Kinnarot and Dead Sea basins developed (e.g. [7, 9]). Water-bodies, mainly freshwater to brackish lakes [12, 13] filled the basins and valleys in northern Israel depositing sequences of sediments that comprise the Bira and Gesher Formations (described below) [7, 14]. The transition from a broad drainage system that flowed over a “prairie”- type landscape to a “basin” and “around lakes” environment had a profound impact on the forthcoming ecological-evolutionary development of the region [15].

In this paper, we report the first evidence for terrestrial mammal in the late Miocene sedimentary sequence of the Bira Formation at Hagal Stream, located at western margin of the Jordan Valley, in the eastern Lower Galilee area (Fig 1B). The Bira Formation of Tortonian age (~ 10.0–7.0 Ma) [11] comprises a sequence of sediments (limestones and dolomites with marls, gypsums and halite) [16] that were deposited from the lake’s fresh-brackish waters (e.g. [14, 16]). Continental fauna of that period is known from the Mediterranean basin, though hardly from the southern Levant. We describe the deposition environments of the Bira Formation at the Hagal Stream section, present the megafauna fossils from this section and discuss the implications of the discovery of the mammal bone for the evolution of terrestrial late Neogene fauna in the vicinity of Israel.

## 2. The Tortonian-Messinian periods in the circum-Mediterranean

During the late Miocene–early Pliocene times (~ 10 to 5 Ma) the morphological basins and the lowlands that extended over the areas of the Yizre’el Valley, lower Galilee, Kinnarot Basin and Jordan Valley (Fig 1B) were occupied by lacustrine-estuarine and palustrine water-bodies that deposited sequences of carbonates (limestones and dolomites), marls, gypsum and halite (in the Kinnarot Basin only) (e.g. [7, 14, 16–18]). These sedimentary sequences comprise the Bira and Gesher Formations of Tortonian (~10 to ~ 7 Ma) and Messinian-Zanclean (~ 7 to ~5 Ma) times, respectively [11]. During this time-interval marginal lagoons and lacustrine water-bodies developed in the circum-Mediterranean area (e.g. the Pannonian Basin, [19–21]). During the Tortonian period (~11.6 to 7.2 Ma) the Mediterranean was connected to the Atlantic Ocean via several gateways off southern Spain and northern Morocco [22]. Later, during the Messinian period (~7.2 to 5.3 Ma) the Mediterranean Sea became progressively isolated from the Atlantic Ocean, generating widespread precipitation of evaporites (~5.9 to 5.6 Ma), massive salt deposition (~5.6 to 5.5 Ma) and a dramatic sea level lowering (~5.5 to 5.3 Ma) during the Messinian Salinity Crisis (MSC) (e.g. [23–24]). The MSC was abruptly ended at the early Pliocene (~5.3 Ma) by catastrophic flooding (the Zanclean transgression). The Mediterranean returned to open marine conditions [25]. All these processes had their impact on the environmental history of the circum-Mediterranean areas and the development of the regional fauna before the onset of the tectonic movements that caused regional uplifts in the marginal areas of the Mediterranean and the onset of the Ice Ages in the Pliocene-Pleistocene [15, 26–28].

## 3. The late Miocene Bira and Gesher Formations

A sequence of sedimentary rocks termed the Bira and Gesher Formations was deposited in lacustrine water bodies that occupied during the late Miocene-early Pliocene periods the areas of the Lower Galilee, Yizre’el Valley and the Jordan Valley. The formations were deposited between the time of eruption of the Lower Basalt and the Cover Basalt Units (between ~10 and ~ 5.3 Ma) (e.g. [7, 14]. The depositional regime of the Bira and Gesher Formations was mostly lacustrine. This regime was occasionally interrupted by transgressions of Mediterranean Sea waters (e.g. [7, 14, 16, 29, 30]). Coinciding with the existence of the lakes in the Lower Galilee, Yizre'el and Jordan Valley margins, evaporitic water-bodies were developed in two unique tectonic basins that were associated with the tectonic activity along the Dead Sea Transform Fault [1, 31 and references herein]) and the Kinnarot Basin (Fig 1B and 1C) where the Zemah-1 borehole recovered ~ 4.25 km of carbonates, marls, gypsum, halite and basalts [32] and the Dead Sea Basin, where boreholes recovered several thousand meters of fluvial, lacustrine and evaporitic sediments comprising the Sedom Formation [29]. Thick sequences of salt from marine origin were deposited in the Kinnarot and Dead Sea Basins, respectively (e.g. [29, 31–33]).

**Fig 2:**
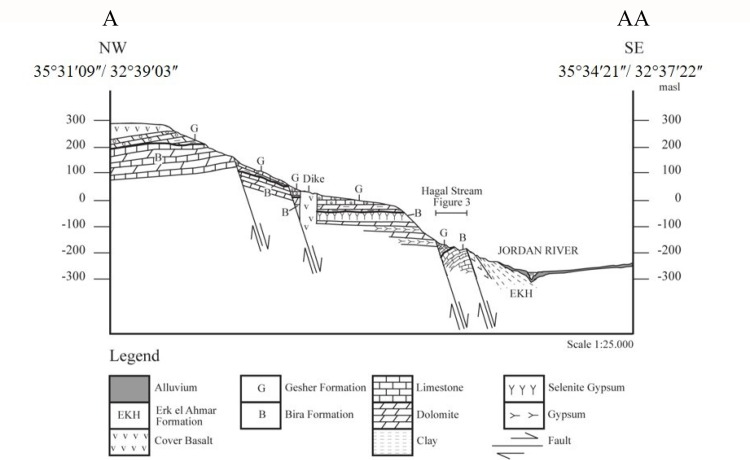
NW-SE cross section A-AA along the Hagal Stream (modified after [17]).

The Tortonian (~10–7.2 Ma) Bira Formation, where the mammal remains were discovered, comprises limestones, dolomites, marls, gypsum sediments and basaltic rocks (Figs 1C and 2) and halite at the Kinnarot Basin. The lacustrine water bodies of the Bira Formation comprehend shallow water-bodies that extended from the western border of the Lower Galilee in the west to the Golan Heights in the east including the area of the Pleistocene–Modern Sea of Galilee [7, 16]. The surface levels of Lake Bira changed frequently allowing the setting of evaporitic conditions and formation of dolomites [36]. The lacustrine regime was interrupted by two prominent marine (Mediterranean) ingressions, at the base and the top of the Bira Formation (e.g. [7, 14, 16, 30]).

## 4. The Bira and Gesher Formations at the Hagal Stream section

**Fig 3:**
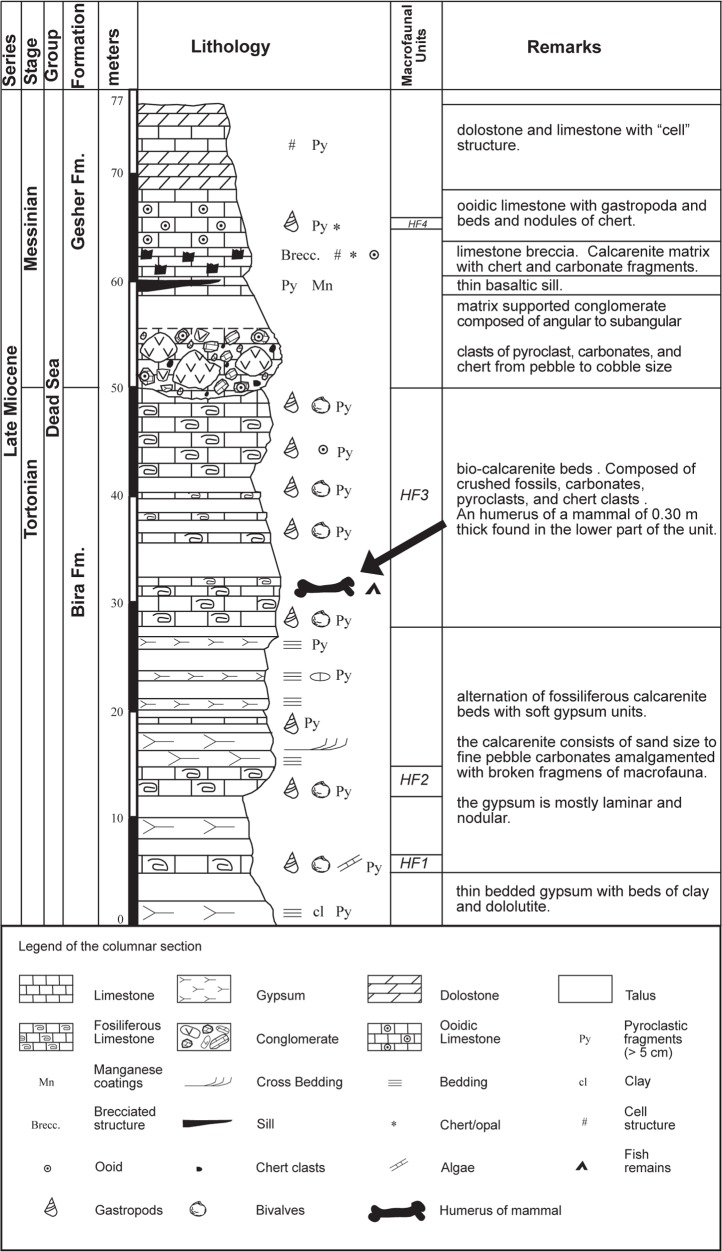
Lithostratigraphy of the Hagal Stream Section. The top of the section is at coordinates 35°33ʹ01ʺ, 32°37ʹ57ʺ and elevation 140 meters below sea level. Legend indicates the lithology and main features symbols observed in the columnar section.

**Fig 4:**
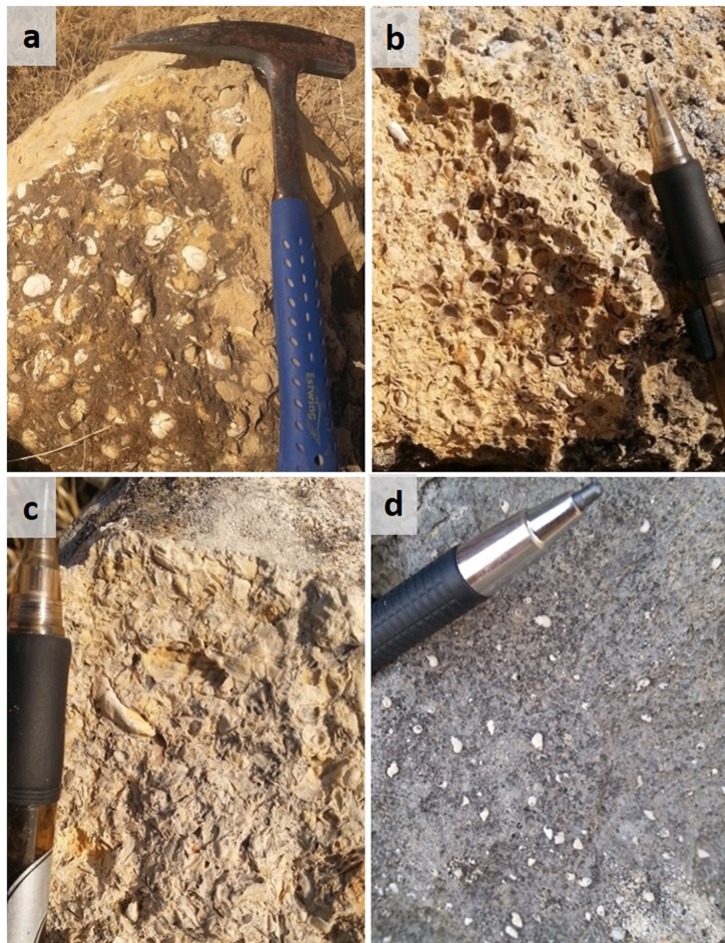
The molluscan fossil assemblages of the Hagal section. (a) Loosely packed moderately fragmented bivalves (mainly Unionidae) in their original shells from the upper part of unit HF1; (b) Densely packed scarcely fragmented Neritidae gastropods (*Theodoxus* spp.), preserved as external printings or inner molds, comprising the monospecific assemblage of unit HF2; (c) Densely packed extensively fragmented bivalves and gastropods of unit HF3, Dreissenidae mold and Melanopsidae imprint are identifiable; (d) Sparse-loosely packed complete Assimineidae gastropods, comprising the monospecific assemblage of HF4.

**Fig 5:**
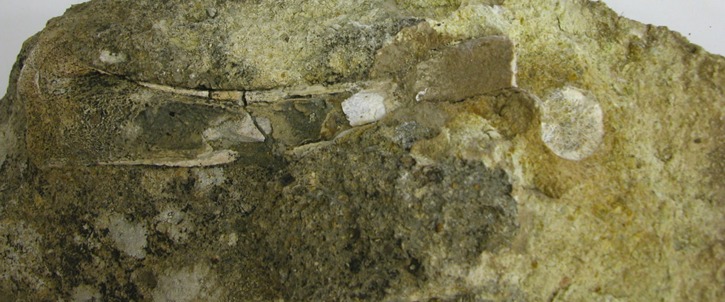
The block from Hagal section (unit HF3) in which the bone was found. The bone was embedded into the sediment. Notice the severe erosion of the shaft and the proximal epiphyses.

Here, we focus on the Hagal section of the Bira Formation. The section is located at the eastern side of Hagal Stream, at the western escarpment of the Dead Sea Rift (Fig 1B and 1C). The section forms a recumbent fold, part of a half-dome located between two shear zone faults of the western Jordan Valley margin (Figs 1C and 2). The layers dip sharply (~ 70 to 40°) from SE to NW (Fig 1C and Fig 2). The Bira Formation at the Hagal section comprises about 50 meters of fossiliferous carbonates, which form benches in the landscape, and soft units of gypsum, which are mostly covered by soils or talus (Fig 3). The base of the formation is not exposed. The fossiliferous benches contain gastropods and bivalves within calcarenite matrix with carbonate cement (Fig 4 and see below). Some beds consist of fragments to pulverized fossils, suggesting high water energy conditions. On one of these benches, a mammal bone (humerus) (Figs 3 and 5) and a fish jaw were found. Gypsum beds comprise nodular, thin bedding and ripple marks structures. The contact between the Bira and Gesher Formations is erosive. The base of the Gesher Formation is not exposed at the Hagal section. The lower part of the formation comprises a polimictic conglomerate that is overlain by ooidic limestones, limestone breccia and beds and nodules of chert. The top of the section consists of soft limestones and dolostones (Fig 3).

## 5. The macrofaunal assemblage at Hagal section

### 5.1 General description

The macrofaunal remains at Hagal section include gastropods and bivalves of freshwater origin; some of them tolerate or prefer brackish conditions. Neritidae (*Theodoxus* spp.) and Unionidae are the most abundant gastropods and bivalves, respectively. The Bira Formation is divided into three units (HF1-3), according to the macrofaunal assemblages described below. Another unit (HF4) appears in the above-lying Gesher Formation (Table 1).

**Table 1:** The count data for the Hagal section macrofaunal assemblages.

*HF1*			
Taxa No.	Family name	No. of counted individuals	Abundance (%)
1	Neritidae	133	47
2	Unionidae	30	11
3	Cochliopidae	45	16
4	Bithyniidae	27	10
5	Melanopsidae	17	6
6	Thiaridae	25	9
7	Dreissenidae	2	1
8	Planorbidae	1	0
9	Cyrenidae or Sphaeriidae	1	0
10	Potamidae	1	0
	TOTAL	282	
*HF2*			
Taxa No.	Family name	No. of counted individuals	Abundance (%)
1	Neritidae	155	97
2	Melanopsidae	4	3
	TOTAL	159	
*HF3*			
Taxa No.	Family name	No. of counted individuals	Abundance (%)
1	Neritidae	89	44
2	Melanopsidae	77	38
3	Dreissenidae	23	11
4	Thiaridae	10	5
5	Unionidea	4	2
	TOTAL	203	
*HF4*			
Taxa No.	Family name	No. of counted individuals	Abundance (%)
1	Assimineidae	114	100
	TOTAL	114	

#### HF1

The lowermost beds of the exposed section comprise calcarenite with loose-densely packed shells (following shells close-packing definitions by [37]). The assemblage characterizes by 10 freshwater mollusk families, dominated by gastropods (88% abundance). Neritidae (*Theodoxus* spp.) is the most abundant taxa, but with relatively low dominance of 47%. Other gastropod families include Cochliopidae, Bithyniidae, Thiaridae, Melanopsidae, and Planorbidae. Bivalves, mainly Unionidae, show increase in abundance towards the upper part of HF1, where they appear moderately fragmented in their original shells (Fig 4A).

#### HF2

This unit comprises brown-reddish soft bio-calcarenite of fine size with 2.5% pyroclasts of silt and sand size and densely packed shells. The assemblage is characterized by very low species richness of two gastropod families, with very high dominance of Neritidae gastropods (*Theodoxus* spp.), comprising 97% of the assemblage. Most of the fossils in this nearly monospecific assemblage appear as external printings, sometimes with preserved colored sculptures, some appear as inner molds (Fig 4B). Fragmentation degree is low to moderate.

#### HF3

The upper 25 meters of the Bira Formation comprise light gray to light brown soft bio-calcarenite beds that characterized by fine grain size, with 2.5–5% pyroclasts of silt to fine pebble size, and contain mainly crushed shells (Fig 4C). Occasionally, numerous specimens appear unbroken in the surrounding fragments, especially of the epifaunal families Neritidae, Melanopsidae and Dreissenidae (Fig 4C). This could be the result of different shell strengths, sizes, thicknesses and shapes affecting the degree of resistance to breakage [38]), or imply a complex taphonomical history of recolonization by next generations (e.g. [39]). The assemblage of the recovered fossils from the crushed surrounding of unit HF3 is characterized by median species richness of 5 families, with low dominance of the most abundant taxa, which is of Neritidae family, comprising 44% of the assemblage.

#### HF4

A single fossilized bed is identified in the Gesher Formation. The bed consists of a brown highly indurated ooidic limestone, with ooids of fine sand to silt size, and sparse-loosely packed shells. The assemblage is comprised entirely from brackish minute gastropods of the Assimineidae family, comprising 100% of the assemblage (genus *Assiminea*, Fig 4D).

### 5.2 Environmental conditions

The paleontological and lithological characteristics of the Bira Formation at the Hagal section (Fig 3 and Table 1) indicate that the water-body was strongly influenced by salinity changes. Variations in the community structure between assemblages, expressed by the ecological parameters of species richness and dominance, indicate variations in the environmental stress levels (e.g. [40–42]). Monospecific assemblages are rare and commonly result from opportunistic species taking advantage of unusual environmental conditions that would be stressful for most species (e.g. [40, 43–44]). The variations in the species richness (see Table 1), showing a major decrease from the relatively rich assemblage of unit HF1 to the nearly monospecific assemblage of unit HF2, indicates that environmental stress levels increased. Then, the increase in species richness from HF2 to the relatively rich assemblage of HF3, indicates environmental stress levels decreased again. The same pattern is presented by the variations in the abundance of the dominant Neritidae gastropods, from less than 50% of the assemblage in HF1, to complete dominance in HF2, and back to less than 50% of the assemblage in HF3. These shifts in dominance, together with the highly euryhaline nature of these gastropods [45–46], strongly suggest salinity was the main factor controlling community structure [40, 47]. The alternating lithology, between carbonatic rocks rich with fresh-brackish fossils and gypsum layers, support this conclusion and further imply the influence of salinity variations in the investigated section, from periods of fresh-brackish water to periods of hypersaline conditions.

The taphonomic characteristics of the macrofaunal assemblages of the Bira Formation in the Hagal section indicates that changes in water depths and energy levels were dominant factors in assemblage formation. The fragment shapes and sizes and the lack of predation traces in unit HF3, indicate the extensive breakage was caused by washing and pounding of the shells on a high-energy shore [38, 48, 49] The sharp taphonomic shift with time from low-medium fragmentation (units HF1, HF2) to massive breakage (unit HF3), implies a shift from relatively deep and quiet conditions to shallower, more energetic conditions very close to the lake’s shorelines. The disappearance of gypsum beds (Fig 3) and the finding of the mammalian bone (see below) in unit HF3 also support the suggested decrease in water depth and setting near the shorelines of the lake.

The complete monospecific character of unit HF4 from the Gesher Formation at the Hagal section imply high levels of environmental stress (e.g., [43–42]. In lack of other fossilized bed in the Gesher Formation., it is difficult to fully understand the general environmental conditions during this period. However, the minute gastropods that comprise this entire assemblage, *Assiminea* sp., is known to inhibit brackish water bodies [50–51], therefore the high environmental stress indicated by the monospecific appearance can possibly be related to salinity, similarly to the Bira Formation. The appearance of dolomite layers and disappearance of gypsum layers in the Gesher Formation can point to relatively less hypersaline conditions during this deposition period, compared to the Bira Formation.

### 5.3 Comparison with other sections in the region

An important conclusion so far is that variations in salinity characterized Lake Bira at the Hagal Stream area, from periods of fresh to brackish conditions during which carbonate rock rich with macrofauna were deposited, to hypersaline-evaporated conditions during which gypsum beds were deposited. Similar conditions also prevailed in other areas, for example the Hurvat Ze’ev section, located east to Hagal section, at the Tabor Stream area (Fig 1B). The macrofaunal assemblage of the Hurvat Ze’ev section is mostly fresh-brackish water fossils, with similar taxonomic family list as the Hagal section. However, the most abundant taxa in these two sections is different, with Thiaridae family (*Melanoides* spp.) as the dominant taxa in Hurvat Ze’ev, and Neritidae family (*Theodoxus* spp.) as the most dominant taxa in Hagal section. The more euryhaline and opportunistic nature of the Neriitida family, compared to the Thiaridae family, points to increased salinity-stressed conditions in the Hagal section.

Marine assemblages comprise 10% of the fossiliferous Hurvat Ze’ev section, and are distributed in five beds along the section that represent transgression events of Mediterranean Sea water. These marine assemblages are nearly monospecific, comprising almost entirely from small-sized euryhaline bivalves of the Veneridae family (*Venerupis* sp. and *Paphia* sp.), indicating high levels of environmental stress during these saline episodes [52]. Furthermore, the Hurvat Ze’ev section characterizes by appearance of relatively thin dolomite beds, which are related to periods of significant evaporation and interpreted as related to climatic changes toward arid periods [36]. Thus, it seems that the evaporitic conditions were more intense when moving east to the Hagal area, enabling the deposition of gypsum beds. Further to the west, at Migdal Ha’Emeq section of the Bira Formation. (Fig 1B), a dramatic change is observed in the macrofaunal assemblages, reflected in the distribution of fossiliferous beds and the species composition. Only two beds are rich with macrofauna, indicating that for most of the time the conditions on the bottom floor were unsuitable for colonization. These two assemblages are comprised entirely from marine species, and their community structure properties indicate good ecological conditions. The rest of the Migdal Ha’Emeq section is composed of carbonate rocks, mainly limestones. The disappearance of the fresh-brackish community towards the west indicate more saline conditions and the rich marine assemblages imply periods of major sea water intrusions. The connection to the Mediterranean is decreasing to the east, through the ecologically-stressed assemblages in the Hurvat Ze’ev section, and to the entirely fresh-brackish assemblages of the Hagal section. The solution that deposited the gypsum beds in the Hagal section might have originated from these marine transgression events.

### 5.4 The mammalian bone

**Fig 6:**
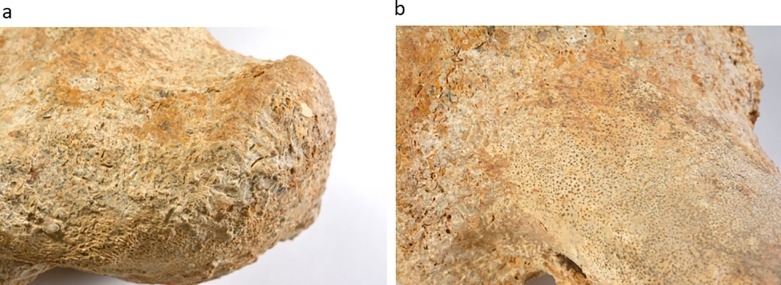
Close up of the bone surfaces: (a) sediment compacted still adherent to the bone surface; (b) black spots probably due to organic contamination.

In a block that fell from unit HF3 (Fig 3), the outlines of a limb bone were observed. The bone was embedded in the sediment and the exposed part was already eroded when found (Fig 5). It first seemed that the compaction in the crushed mollusks had deformed the general outline. Being such a rare find, apparently the first known mammalian limb bone from the late Miocene in the area, we invested every possible effort to expose the limb. Combinations of diluted acid solution and mechanical cleaning were required in order to finally release the bone from the sediment. However, the complete exposure of the bone outer surface was not entirely possible as the sediment is "imbedded" in the bone (Fig 6A). Microscopic observation supported the necessary final observation for the outer shape of the bone. Black miniature spots probably due to organic contamination were observed over the bone. This possible remnant of organic material is under study (Fig 6B). Taphonomic observations are partially masked by the embedded sediment coverage. The erosion of the shaft is probably due to water wave movement along the shores of the lake.

**Fig 7:**
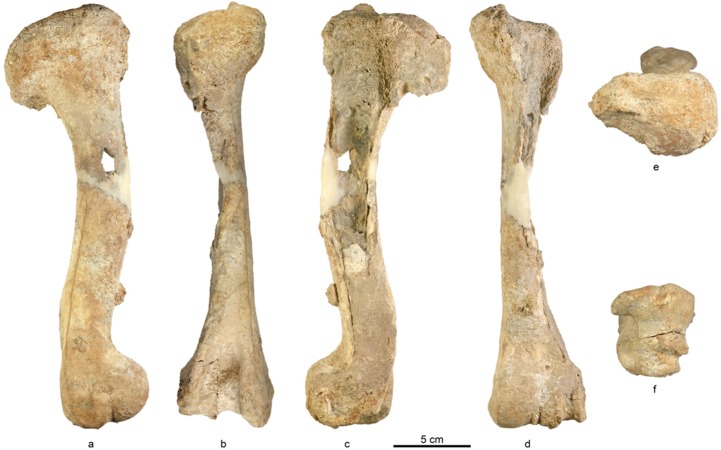
Various aspects of the humerus from Hagal section (unit HF3): (a) medial view; (b) posterior view; (c) lateral view; (d) anterior view; (e) proximal head; (f) distal condyle.

*Description of the bone (NGM1)*: almost complete left humerus of an adult animal (proximal epiphysis fused). The proximal head is not complete and the lateral tuberosity is missing (Fig 7C–7E), the shaft along the lateral view is missing (Fig 5 and Fig 7C), the distal end is almost complete, though parts are missing from the lateral condyle (Fig 7B and 7C). In the absence of the shaft (e.g., missing the area of the deltoid tuberosity) and most of the proximal part (e.g., lateral tuberosity and inter tuberal groove) the possibility to assign it to a species is limited.

Following the characteristics of the distal part described by [53, Vol. I, Planche XXXVI, 6; Vol. II, p.30], medial valley is situated high in comparison with the external condyle, the external lip is acute and the internal condyle surpasses it, as is typical in cervids. We suggest that this humerus is similar to a cervid. However, we are aware of the problematic of identifying a genus in the absence of teeth and cranial parts.

Family Cervidae Gray, 1821

Cervidae gen. and sp. indet

Humerus (left, NGM1), Hagal Stream, Unit HF3, Bira Formation, late Miocene (Tortonian).

*Measurements of the bone (NGM1)*: Humerus Cervidae gen. and sp. indet

GL (total length on anterior view) - 25.348 mm

Bp (breadth of the proximal end)—ca. 82 mm

BT (breadth of the distal condyle) - 59.85 mm

Bd (breadth of the distal end) - 58.40 mm

SD (smaller breadth of diaphysis)—ca. 34.75 mm

Tortonian localities are rich in terrestrial vertebrate animals from small herbivores to large proboscideans. Tortonian mammals are known from the Kefraya and Zahle Bekaa Valley in Lebanon [54–55]. [56–57] reported the presence of a proboscidean tooth from sediments of the Gesher Formation at a site located northeast of the Sea of Galilee, near Sheikh Ali [57 p.322].

Fossil cervidae are part of the faunal components of early Miocene onwards in Eurasia. The nearest ancestors of the recent taxa were identified in the late Miocene, though there is a dispute about the relation between the ancestor's origins. Many species were identified based on the antler and dentition [58 and references herein]. Cervids were reported from many late Miocene localities from the Levant and the Mediterranean realm, to mentioned just a few: the areas of Maragheh Iran [59], many sites from Turkey [60] Greece [61–62], Italy[63] and Spain [64].

This sole limb is just a testimony of a probable rich fauna invisible due to post depositional processes that buried most of the Tortonian localities in the study area. In the absence of antler and teeth how can we better identify the current element?, and can we speculate more on the basis of the available humerus properties? The length of the element (GL) excludes the presence of any medium cervid as is much larger than the local *Dama* (*Dama mesopotamica*) that probably prevailed from the early Middle Pleistocene onward [65]. [66] suggested to examine the mid-diaphysis properties of long bones by looking at the mid-diaphyseal properties of limb long bones, characterizing the thickness of the bone walls, but actually found little correlation between the humeri and the allometry of cervidae species. As yet, a certain thinning of the cortical wall and bending was observed on larger species as can be noticed on the *NGM1* humerus (Fig 7).

The Tortonian paleo-environment of local fresh water-bodies offers ambient conditions to terrestrial species such as large size cervids. The paleontological and genetic data refer to the radiation of old world deer [67] during the late Miocene. Earlier evidence points towards eastern Asia as the region of origin of the group *sensu lato* [68 and references herein]. Geographical buries prevented continues dispersal, though larger cervids with greater territories and flexible dietary needs have advantageous in dispersal. Along the dispersal route from East Asia we can witness sites in Iran [59] crossing through various water-bodies into the studied area.

## 6. Summary

The recent finding of an artiodactyls element, probably of a cervid, from the late Miocene section of the Bira Formation contributes to the local paleoenvironmental reconstruction. The salinity of the water-body (Lake Bira) was mostly fresh, as indicated by the molluscan identification. A variety of organisms of Tortonian times undoubtedly used this lake as an important water source. The mammalian bone was found in unit HF3, in which a major decrease in water depth and increase in water energy is indicated by the extensive fragmentation and pulverizing of the fossils. The cervid probably died for some reason while drinking water from the lake shorelines. Thus, the finding of the mammalian bone reinforces the reconstruction of the depositional environment as shifting to shorelines conditions. The strong wave action rolled the outer surface of the bone and the changing shore water regime caused the sediment compaction. Further survey in the area might reveal auxiliary exposure richer in vertebrate fauna as expected from Tortonian localities.

The development of fresh water-bodies during the late Miocene in the Levant and circum-Mediterranean area opened the route for the ancient deer expansion westward.
